# Good syndrome with cytomegalovirus hepatitis: successful resection of Thymoma: a case report

**DOI:** 10.1186/s13019-020-01187-y

**Published:** 2020-06-15

**Authors:** Sho Isobe, Atsushi Sano, Hajime Otsuka, Yoko Azuma, Satoshi Koezuka, Takashi Makino, Takashi Sakai, Takafumi Ito, Tadashi Maeda, Kozue Ejima, Sakae Homma, Akira Iyoda

**Affiliations:** 1 Division of Chest Surgery, Toho University School of Medicine, 6-11-1 Omori-nishi, Ota-ku, Tokyo, 143-8541 Japan; 2 Department of Respiratory Medicine, Toho University Omori Medical Center, Tokyo, Japan; 3 Department of General Medicine and Emergency Care, Toho University School of Medicine, Tokyo, Japan; 4 Department of Surgical Pathology, Toho University School of Medicine, Tokyo, Japan

**Keywords:** Good syndrome, Thymoma, Cytomegalovirus hepatitis, Thymectomy

## Abstract

**Background:**

Good syndrome is a rare condition, manifesting as immunodeficiency due to hypogammaglobulinemia associated with thymoma. Herein, we present a patient with Good syndrome whose thymoma was resected after treatment of cytomegalovirus hepatitis.

**Case presentation:**

The patient was a 45-year-old woman presenting with fever, cough, and nasal discharge, and was diagnosed with thymoma and hypogammaglobulinemia. She subsequently developed cytomegalovirus hepatitis that was treated by immunoglobulin. After resolution of the hepatitis, she underwent thymectomy through a left anterior thoracotomy. Her postoperative course was uneventful, and while receiving ongoing immunoglobulin therapy, she has been doing well without signs of infection.

**Conclusions:**

Management of infections is important for patients with Good syndrome. To minimize the risk of perioperative infection, we should take care while planning the surgical approach and procedure.

## Background

Good syndrome, the condition of adult-onset immunodeficiency associated with thymoma, is quite rare, occurring in only 0.2–6% of thymoma patients [1, 2]. Patients with typical Good syndrome show hypogammaglobulinemia and B-cell depletion. Preventing infection improves the prognosis of patients with Good syndrome, and repeated gamma globulin therapy is considered necessary [3, 4]. Herein, we report a patient with Good syndrome who underwent successful resection of her thymoma through a left anterior thoracotomy and received preoperative gamma globulin therapy subsequent to treatment for preoperative cytomegalovirus hepatitis.

## Case presentation

**Fig. 1:**
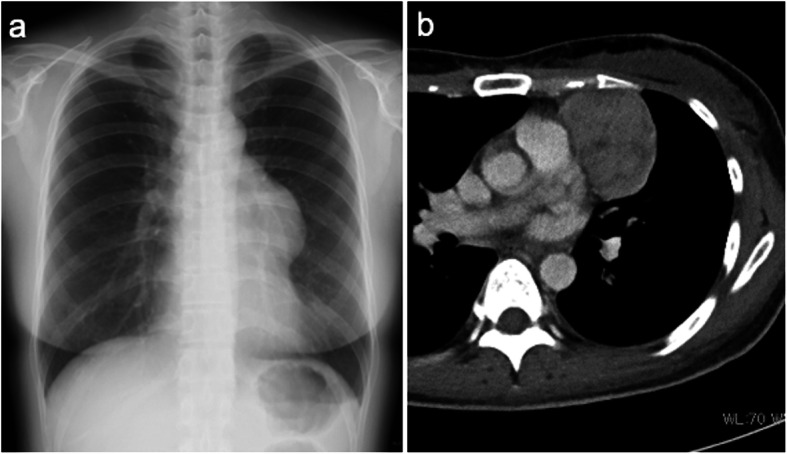
Chest X-ray and Computed tomography on diagnosis. Chest X-ray showing a mediastinal tumor protruding into the left chest cavity (**a**). Chest computed tomography scan showing a well-defined 61 × 45-mm tumor (**b**)

The patient was a 45-year-old woman who was referred to a nearby clinic for fever of 38 °C, cough, and nasal discharge. Although she was treated with antibiotics, her signs were not improved. Chest X-ray and computed tomography showed a 61 × 45-mm anterior mediastinal tumor (Fig. 1). Positron emission tomography scan showed 1.8-fold greater uptake than the maximal standardized uptake value in the tumor. A blood test revealed a serum immunoglobulin G level of 239 mg/dL (normal range 870–1700 mg/dL), serum immunoglobulin A level of 24 mg/dL (normal range 110–410 mg/dL), and a serum immunoglobulin M level of 26 mg/dL (normal range 46–260 mg/dL). She was referred to our hospital for further examination and treatment for the anterior mediastinal tumor and hypogammaglobulinemia. The histopathological diagnosis of a CT-guided biopsy specimen was type AB thymoma based on the World Health Organization classification, leading to the diagnosis of Good syndrome.

While undergoing diagnostic workup, the patient developed sudden deafness that was treated by corticosteroids. She then became febrile with worsening liver function, showing a serum aspartate aminotransferase level of 127 U/L and a serum alanine aminotransferase level of 132 U/L. She developed serum cytomegalovirus antigenemia, and altogether, the findings were diagnosed as cytomegalovirus hepatitis due to hypogammaglobulinemia. She received 15 g of immunoglobulin and ganciclovir with subsequent improvement in her liver function, with normal serum levels of aspartate aminotransferase and alanine aminotransferase. Her serum cytomegalovirus antigenemia was undetectable 2 weeks after initiation of antiviral therapy.

**Fig. 2:**
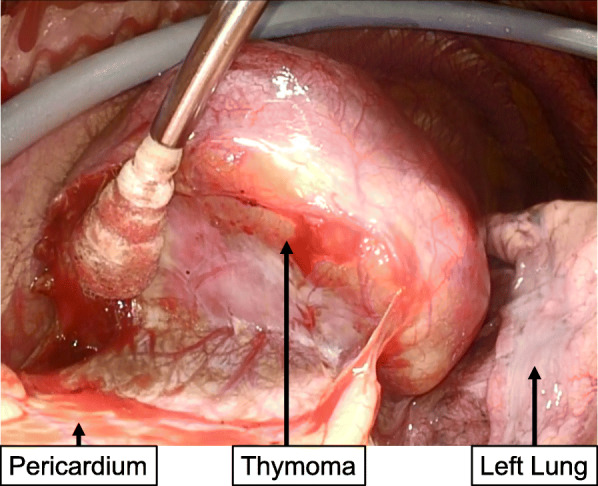
Intraoperative view. The thymoma did not invade surrounding tissues and was easily dissected

**Fig. 3:**
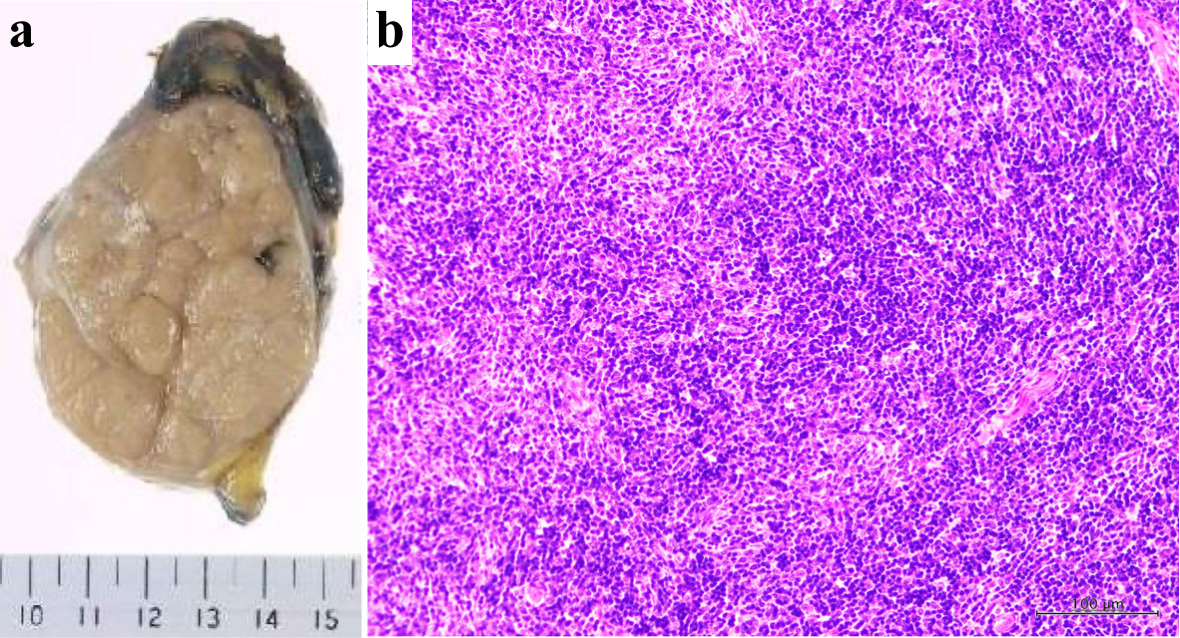
Operative specimen. (**a**) Macroscopically, the tumor was encapsulated grayish-white mass with a size of 80x42x63mm. (**b**) Microscopic picture. Hematoxylin and eosin stain 200X. The tumor was consist of a variable mixture of lymphocyte-poor type A-like components and lymphocyte-rich type B-like components

After her cytomegalovirus hepatitis improved, the patient underwent surgical resection for thymoma. Because she was immunocompromised, we performed a video-assisted left anterior thoracotomy with an 8 cm skin incision instead of a median sternotomy to minimize the risk of a perioperative infection (Fig. 2). We administered immunoglobulin twice before surgery, and thymectomy was performed 3 months after the diagnosis of cytomegalovirus hepatitis. The postoperative course was uneventful without signs of infection, and the patient was discharged 10 days after the surgery. Macroscopically, the tumor was encapsulated grayish-white mass with a size of 80x42x63mm (Fig. 3a). Pathological diagnosis showed type AB thymoma (Fig. 3b).

**Fig. 4:**
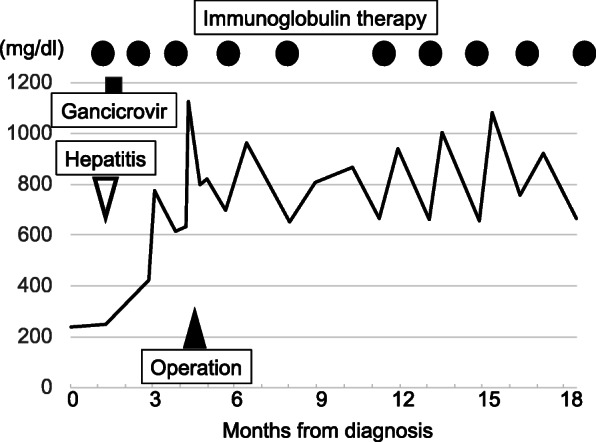
Transitions in serum immunoglobulin G levels. Black circles indicate intravenous immunoglobulin therapy. Black triangle indicates the surgical procedure, and white triangle indicates the onset of cytomegalovirus hepatitis infection. A black square indicates the duration of ganciclovir treatment

The patient remains alive without recurrence of thymoma for 26 months. Her hypogammaglobulinemia has persisted, and she has undergone regular administration of immunoglobulin therapy (Fig. 4). She has not developed signs of infection since the immunoglobulin therapy was initiated. Sudden deafness was not improved by corticosteroids. Six months after thymectomy, the cochlear implant was performed for deafness.

## Discussion

Good syndrome is characterized as a combination of thymoma and hypogammaglobulinemia. In patients with Good syndrome, hypogammaglobulinemia often results in bacterial and viral infections, which are sometimes fatal [3, 4]. Therefore, the control of infection is important in patients with Good syndrome.

Bacterial infections are the most frequent in patients with the Good syndrome, followed by viral infections, with cytomegalovirus infection being the most frequent viral infection [4]. Cytomegalovirus duodenoenteritis and retinitis have been reported in patients with Good syndrome [5, 6]. According to these previous reports, ganciclovir was an effective treatment.

To the best of our knowledge, this is the first report of cytomegalovirus hepatitis in a patient with Good syndrome. Cytomegalovirus hepatitis sometimes occurs in immunodeficient patients with human immunodeficiency virus infection or undergoing organ transplantation. Cytomegalovirus infection can relapse after improvement due to ganciclovir treatment, if the patient’s immunodeficiency worsens. In our case, the use of corticosteroids in addition to hypogammaglobulinemia induced relapse of cytomegalovirus infection. Therefore, we administered immunoglobulin therapy before and after the patient’s thymectomy to prevent relapse of hepatitis during the perioperative period, and long after the surgery.

The prevention of surgical site infection during the perioperative period is also important. We administered immunoglobulin therapy before the operation and after the operation prophylactic intravenous antibiotics for a week. In addition, we performed a video-assisted left anterior thoracotomy for a left anterior mediastinal tumor instead of a median sternotomy. Mediastinitis is a complication after sternotomy, and postoperative mediastinitis was previously reported in a patient with Good syndrome who underwent thymectomy through a median sternotomy [7]. Therefore, we avoided performing it. Although a thoracoscopic approach is another option, we performed a thoracoscopy-assisted lateral thoracotomy because the tumor diameter was at least 6 cm. It was recently reported that thymectomy alone is appropriate for patients with stage I thymoma [8]. To minimize the risk of postoperative infection, we think thymectomy through a lateral thoracotomy is reasonable for patients with Good syndrome with stage I thymoma.

## Conclusion

Management of infections is important for patients with Good syndrome. To minimize the risk of perioperative infection, we should take care while planning the surgical approach and procedure.

## Data Availability

Not applicable.
